# Supplementary material for: Dissociation of tic generation from tic expression during the sleep-wake cycle
Source: iScience. 2021 Mar 31;24(4):102380. doi: 10.1016/j.isci.2021.102380 (PMC8081921; doi:10.1016/j.isci.2021.102380)
Supplement: Document S1. Transparent methods [file mmc1.pdf]

iScience, Volume *24*

Supplemental information

Dissociation of tic generation from tic expression during the sleep-wake cycle

Esther Vinner Harduf, Ayala Matzner, Katya Belelovsky, and Izhar Bar-Gad

Transparent Methods

Animals

Eleven adult rats (Long-Evans, females) weighting 269±19 g (mean ± SD) were used in this study. The rats had access to food and water *ad libitum* and were maintained under controlled temperatures and a 12 h light/dark cycle. All procedures were approved and supervised by the Institutional Animal Care and Use Committee and adhered to the National Institutes of Health Guide for the Care and Use of Laboratory Animals and the Bar-Ilan University Guidelines for the Use and Care of Laboratory Animals in Research. This study was approved by the National Committee for Experiments in Laboratory Animals at the Ministry of Health.

Surgery

An infusion-cannula (stainless steel 30 AWG tube), curved at 90º, was attached to a filled mini-osmotic pump (ALZET pumps, DURECT Corporation), using a flexible polyethylene catheter-tubing (PE-10) and a tubing adapter (CMA Microdialysis). The mini-osmotic pump (9 animals: ALZET Model 2001, volume 200 µl, release rate 1 µl/h, 7 days; 2 animals: ALZET Model 2002, volume 200µl, release rate 0.5 µl/h, 14 days) and the catheter were filled with artificial cerebrospinal fluid (ACSF containing (in mM): 145 NaCl, 15 HEPES, 2.5 KCl, 2MgCl2, 1.2 CaCl2, PH 7.4 with NaOH) or bicuculline methiodide (Sigma-Aldrich) dissolved in ACSF (final concentration: 1 µg/µl for ALZET model 2001 or 2 µg/µl for ALZET model 2002), and then primed in a saline bath at 37 ºC for 4-6 hours prior to the implantation surgery. The surgery included insertion of the pump into a subcutaneous pocket in the rat's back, and implantation of the infusion-cannula into the dorsolateral striatum (infusion target: AP, 1.0 mm; ML, 2.5 mm; DV, 5 mm) (Paxinos and Watson, 2007) to enable ongoing local micro-infusion (Vinner et al., 2017, 2020).

Custom-made movable bundles of 16/32 Formvar-insulated nichrome microwires (25 µm diameter) (Yael et al., 2013) were implanted targeting different basal ganglia nuclei and cortical areas. Only the recording from electrodes targeting the dorsolateral striatum (AP, 0.25 mm; ML, 2.75 mm; DV, 4 mm) and confirmed histologically as being in that location were included in the dataset used for neuronal analysis (N=7 rats). The other animals were only included in the analysis of the behavior.

Experimental sessions

Initial implantation of bicuculline-filled pumps (3 animals) led to tic expression that commenced during the first day after surgery. In the case of an initial ACSF infusion (8 animals), the rats had a control period of 5-7 days followed by a bicuculline pump exchange and subsequent tic expression. Each experimental session, lasting 100±48 (mean ± SD) minutes, included at least one full spontaneous sleep-wake cycle. During the experimental sessions, neurophysiological and kinematic data were recorded continuously while the animal was awake, moving freely, or sleeping in the recording chamber. The striatal neurophysiological signals were recorded in seven rats across eighteen sessions. The kinematic signals were also recorded in seven rats during eighteen sessions. Of these, ten sessions, recorded from three rats, included simultaneous recordings of both neurophysiological and kinematic signals. The striatal neurophysiological signals were recorded using either a wired system (wide band-pass filtered 0.5–10,000 Hz four-pole Butterworth filter; sampled at 44 kHz; AlphaLab SnR, Alpha Omega Engineering) or a wireless system (wide band-pass filtered 1 Hz single-pole to 7000 Hz three-pole Bessel filter; sampled at 32 kHz; Deuteron Technologies). During recordings from the wireless system, kinematic signals were recorded concurrently using a nine-parameter movement sensor covering the X, Y, and Z axes of an accelerometer, a gyroscope, and a magnetometer (MPU 9150, InvenSense), and were sampled at 1 kHz. All the recorded signals were synchronized with a video stream (30/60 frames/s; HCW850, Panasonic), which enabled offline manual assessment of the behavior.

Data preprocessing and analysis

The data recorded during each session were composed of three synchronized signals: video, kinematic sensors, and neural activity. The rats' behavior was extracted from the video stream and was manually classified offline, frame-by-frame, into six behavioral states: quiet waking, sniffing, exploration, grooming, feeding, and sleeping. Motor tics were extracted semi-automatically from the kinematic data. The X-axis of the gyroscope signal was optimal for motor tic identification (as compared to a human expert viewing the video). To compare the quiet waking and sleep states, we sampled the session to obtain two periods of clean and distinct continuous behavior, which lasted 1-3 minutes each. We defined an additional transition period of behavior which

consisted of a continuum from falling asleep that began with eyes closed or half-closed, contained short waking segments and terminated with the continuous sleep period. The dataset analyzed in this manuscript only covers these three types of segments, in each session.

The neurophysiological data were preprocessed offline to extract the local field potential (LFP), the multiunit activity (MUA) envelope, and single-unit spike trains. The LFP was extracted using a low-pass filter (100 Hz 4 pole bidirectional Butterworth filter), and the electrode with the largest signal-to-noise ratio (SNR) was chosen. The MUA was extracted using a band-pass filter (300-6000 Hz 6 pole bidirectional Butterworth filter), and for each electrode, the median of all other electrodes within the same session was subtracted. Then, the MUA envelope was calculated using a Hilbert transform (Moran and Bar-Gad, 2010). The single-unit spike trains were extracted from the band-passed signal using cluster-based spike sorting (Offline Sorter, Plexon). Spike trains presenting unstable waveforms or firing patterns were excluded from the database. The single-spike trains were labeled as one of three neuron types (spiny projection neurons – SPNs, tonically active neurons – TANs and fast spiking interneurons – FSIs) according to their firing rate, firing pattern, and waveform shape. As the number of TANs was small (N=15), the analysis of their data is not presented. All the offline analyses were performed using custom-written MATLAB code (V2017B; MathWorks).

Motor tics and LFP spikes were detected separately from the gyroscope signal and LFP signal, respectively. Objective identification was performed using the SPRING search strategy (Sakurai et al., 2007). This method utilizes the Dynamic Time Warping (DTW) distance and automatically segments input data into subsequences that match template data. For each session, we created a basic template of a motor tic or a single LFP spike using the first 10 notable events recognized manually. This basic template was then expanded into two templates, where each was a variant of the base template. Each of the templates was fed into the SPRING algorithm, resulting in a list of subsequence matches within the session. LFP spikes or motor tics were defined as subsequences shared by both templates. The onset times of the events were identified to align and average multiple events within a single session, and to explore correlation with other signals.

Throughout the figures - * and ** denote $p<0.01$ and $p<0.001$ respectively.
